# Isolating In-Situ Grip and Push Force Distribution from Hand-Handle Contact Pressure with an Industrial Electric Nutrunner

**DOI:** 10.3390/s21238120

**Published:** 2021-12-04

**Authors:** Cederick Landry, Daniel Loewen, Harish Rao, Brendan L. Pinto, Robert Bahensky, Naveen Chandrashekar

**Affiliations:** 1 Department of Mechanical and Mechatronics Engineering, University of Waterloo, 200 University Avenue West, Waterloo, ON N2L 3G1, Canada; c2landry@uwaterloo.ca (C.L.); dploewen@uwaterloo.ca (D.L.); hrrao@uwaterloo.ca (H.R.); bahensky.robert@gmail.com (R.B.); 2 Department of Kinesiology, University of Waterloo, 200 University Avenue West, Waterloo, ON N2L 3G1, Canada; blpinto@uwaterloo.ca

**Keywords:** electric nutrunner, injury prevention, grip force, pistol-grip hand tool, pressure map, push force, optimization

## Abstract

Objectives: Grip force during hand tool operation is the primary contributor to tendon strain and related wrist injuries, whereas push force is a contributor to shoulder injuries. However, both cannot be directly measured using a single measurement instrument. The objective of this research was to develop and test an algorithm to isolate the grip and push force distributions from in-situ hand-handle pressure measurements and to quantify their distributions among industrial workers using an electric nutrunner. Methods: Experienced automobile assembly line workers used an industrial nutrunner to tighten fasteners at various locations and postures. The pressure applied by the hand on the tool handle was measured dynamically using pressure sensors mounted on the handle. An algorithm was developed to compute the push force applied to the handle of an electric pistol-grip nutrunner based on recorded pressure measurements. An optimization problem was solved to find the contribution of each measured pressure to the actual pushing force of the tool. Finally, the grip force was determined from the difference between the measured pressure and the calculated pushing pressure. Results: The grip force and push force were successfully isolated and there was no correlation between the two forces. The computed grip force increased from low to high fastener locations, whereas the push force significantly increased during overhead fastening. A significant difference across the participants’ computed grip forces was observed. The grip force distribution showed that its contribution to total hand force was larger than other definitions in the literature. Conclusions: The developed algorithm can aid in better understanding the risk of injury associated with different tasks through the notion of grip and push force distribution. This was shown to be important as even workers with considerable power tool experience applied significantly more grip and push force than other participants, all of whom successfully completed each task. Moreover, the fact that both forces were uncorrelated shows the need for extracting them independently.

## 1. Introduction

Upper extremity cumulative trauma disorders (UECTD) are a major contributor to lost work hours in manufacturing environments, often resulting from forceful and repetitive tasks [1]. Many injuries are classified as UECTDs, but the most common include tendonitis, tenosynovitis, and carpal tunnel syndrome (CTS) [2,3]. Tendonitis and tenosynovitis are inflammation-based injuries caused by accumulated strain during loading [4] and internal friction, respectively [3], while CTS occurs when the median nerve passing through the wrist is squeezed or compressed [4]. The effects of these injuries can include acute pain, reduced range of motion, and numbness [5].

Risk factors for UECTDs are split into two categories: non-occupational and occupational. Non-occupational factors include age and obesity, while occupational factors include repetitiveness and forcefulness. It is well known that a higher incidence of CTS is associated with forceful exertions of the hand [6], but also with other biomechanical factors, such as task repetition rate and working with vibrating tools [7]. It is important to minimize these factors, as highly repetitive tasks can increase the risk of CTS by 5.5 times and tendonitis by 3.3 times, while highly forceful tasks can increase the risk of CTS by 2.9 times and tendonitis by 6.1 times. Compounded effects of high repetition and force can increase the risk of CTS by 15 times and tendonitis by 29.4 times [2,5].

Task repetition, such as the number of bolts fastened, is easy to quantify and ergonomists continually strive to minimize it. Quantifying the force, specifically the grip force, is far more challenging. Grip force is usually measured on a single axis using a dynamometer. This approach has been widely used to monitor and control the grip force during glove tests, biodynamic response measurements, and many other experiments involving hand-grip simulations [8,9,10]. According to ISO 15230 [11], the orientation of the main grip force is aligned with the z_h_-axis of the biodynamic hand coordinate system defined in ISO 8727 [12]. This is defined as the axis that “passes proximally through the origin and is the long axis of the third metacarpal bone.” However, it has been shown that the maximum grip force on a cylindrical handle is at an average of 78° from the z_h_-axis [13].

The grip force is an important contributor to the overall hand force, but the quantification of grip force during industrial tool operation is challenging. The grip force must be separated from other forces applied to the hand tool, such as push and pull forces, which, by themselves, increase the risk of shoulder injuries [14]. Uniaxial grip and push forces have been separately measured directly using an instrumented split handle and a force plate [15]. Those measurements are independently relevant to quantify the health effects and have been extensively used for many years for studying hand-transmitted vibration [16,17,18,19]; however, they have been difficult to implement in real work environments [15]. Similar custom instrumented cylindrical handles have also been used to report force distributions when gripping, pushing and pulling a cylinder [20], but the results are only applicable to those independent actions. Thus far, there are no direct measurements of the grip and push forces that are applicable to monitoring in-situ tool usage.

There is no clear consensus on which measurements are required to quantify the stress imposed on the anatomical hand-arm system by different hand tools. An attractive alternative to directly measuring the grip and push forces is the measurement of the force distribution on the handle [20], which is easily integrable with the tool itself using thin-film resistive or capacitive flexible sensors. This kind of pressure mapping has provided access to new methods of indirectly measuring grip and push forces, but has also led to different definitions of each [13,21,22]. Nevertheless, grip and push are always defined as force vectors, but with different reference coordinate system definitions. This shows that uniaxial grip and push forces may be inadequate for a complete representation of the hand-arm stress by hand tools, since grip and push forces vary according to the coordinate system that is used. Moreover, the coupling force [23,24], which is the summation of the grip and push forces, will also vary depending on how they are defined, which makes it difficult to understand the contribution of each to the overall hand-arm stress. On the other hand, the hand-handle contact force, which is the integration of pressure over the handle surface [15], is a scalar that is free of a coordinate system, which represents the actual hand stress but does not yield the independent grip and push force contributions.

The goal of this paper is to demonstrate the feasibility of separating the grip and push force distributions from the measured hand-handle force distribution. For this purpose, an algorithm is proposed to decouple the measured force distribution on a handle into distinct grip and push force distributions, in contrast to single force vectors. The algorithm first solves static equilibrium equations to find the resulting push force from pressure map data. Then, a minimization algorithm is used to find the contribution of each force measurement to the push force, thereby extracting the push force distribution. This is then subtracted from the pressure map data to find the grip force distribution on the handle.

The second objective of this study is to demonstrate the usefulness of such force isolation by collecting data on low, high, and overhead fastening tasks with a pistol-grip electric nutrunner, which simulates the range of fastening locations that is typically found in an automobile assembly line.

## 2. Materials and Methods

### 2.1. Measurements

An in situ-study was designed to measure the force distribution applied to a pistol-grip hand tool during a common bolt fastening task at an automobile manufacturing facility. The subject group consisted of five male and four female participants with power tool experience ranging from 6 months to 25 years, all of whom were employed by the same automobile manufacturer. One male and one female participant were left hand dominant; however, both preferred to operate power tools with their right hand. Participant information is summarized in Table 1, where it can be observed that females tended to be shorter and to have a smaller hand span. 

**Figure 1:**
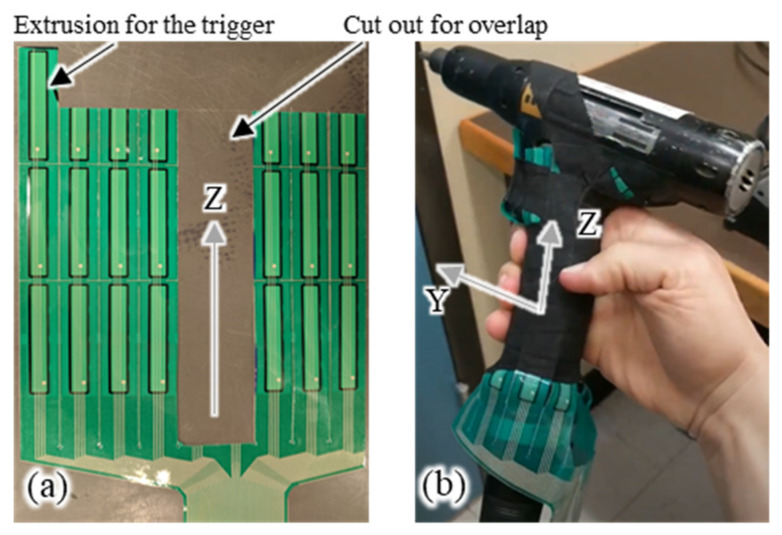
(**a**) Tekscan pressure map trimmed (**b**) and installed on a pistol-grip tool handle.

An electric, handheld, pistol-grip nutrunner manufactured by Atlas Copco (Model ETP ST32-10-10) was used for this study. This is a common direct-current (DC) hand tool that is typically used to tighten fasteners in a manufacturing assembly line. The handle had an ellipsoidal cross-section with minor and major axis lengths of 20 mm and 30 mm, respectively. The tool handle was instrumented with a trimmable Tekscan 9830 pressure mapping sensor (Figure 1) (Tekscan, Boston, MA, USA). This trimmed pressure map contained 72 sensors arranged in 7 columns (or channels), each of which occupied a unique region on the tool handle when wrapped around it. Each rectangular region in a column housed four discrete pressure sensors. There were 10 pressure sensors in each column except for the one overlaid on the trigger, which had 12 sensors (at the extreme left, Figure 1a). The distance between the center of each column was 17.1 mm.

The pressure map was carefully cut in order to adapt to the shape of the tool handle without damaging the electronic circuitry. Each sensor measured a raw output value between 0 and 255 units based on the locally applied pressure. The pressure map was connected to a computer via proprietary hardware and the data were collected using Tekscan’s I-Scan 5.90 software. The pressure map was calibrated using deadweights of known mass before and after the study in order to convert the raw output of the sensor into force in Newtons, since Tekscan does not provide the one-off calibration curve for each sensor.

**Figure 2:**
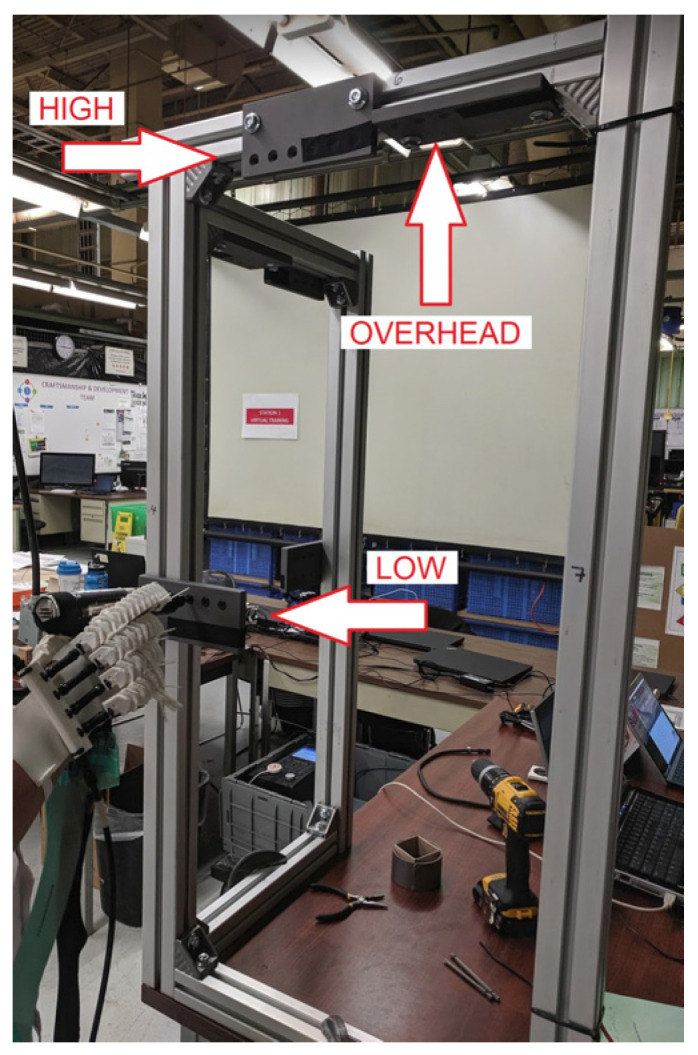
Test frame showing the low, high and overhead locations.

Each participant used the hand tool to fasten three bolts at three different locations and postures on a custom frame (see Figure 2)—low (130 cm horizontal to the ground), high (180 cm horizontal to the ground), and overhead (180 cm vertical to the ground)—the sequence was repeated twice for a total of 18 bolts. These locations simulated the range of fastening locations that is typically found in an automobile assembly line. The torque (5 Nm) and rotation rate of the pistol-grip hand tool were digitally controlled to ensure that every bolt was fastened consistently. Before each trial, data with no force applied to the handle were recorded in order to measure the residual pressure in the sensor that was introduced by mounting the pressure map on a curved surface. Pressure map and tool trigger engagement data based on a binary on-off signal were collected at a sample frequency of 25 Hz. Trigger engagement data allowed for the data to be analyzed only while the tool was engaged, thereby removing the time between fastening bolts as a factor when comparing results.

### 2.2. Data Processing

**Figure 3:**
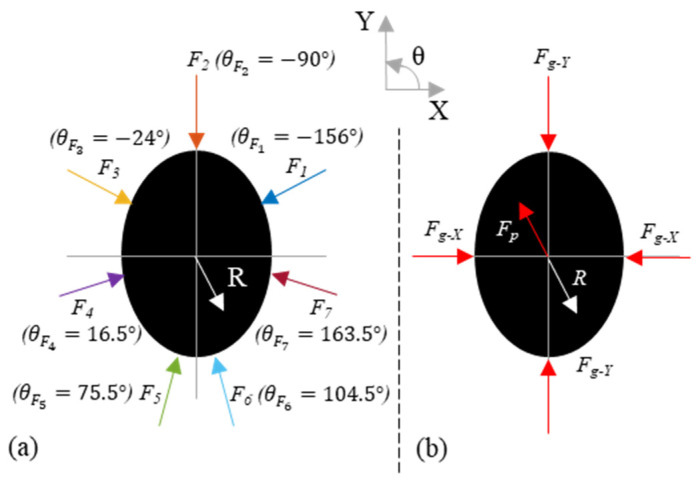
(**a**) Measured forces on the tool handle, and (**b**) extracted grip and push forces by the algorithm.

MATLAB R2019a (MathWorks, Natick, MA, USA) was used to process the data. First, the bias from each sensor was subtracted in order to remove the residual pressure measurement that resulted from contouring the pressure map around the tool handle. Second, the calibration factor was applied to convert the raw pressure values to Newtons. Finally, the measurements from sensors in the same column (Figure 1) were summed in order to obtain the force acting on the tool in the XY plane (handle cross-section plane). The post-processed force measurements acting on the XY plane are shown in Figure 3 with their respective orientations. The orientation of each force measurement was approximated by first computing the position of each sensor’s column based on the arc length of a perfect ellipse starting from the trigger (θ=90°). Second, the angles between the X-axis and each vector perpendicular to the surface for each sensor location were computed.

### 2.3. Optimization

The goal of the optimization algorithm is to find the proportion $w_{i}$ of each force vector ${\vec{F}}_{i}$ contributing to pushing the tool. The algorithm is based on the following hypotheses:

- The pressure on each sensor is uniformly distributed.
- Friction forces on the handle are negligible.
- All forces acting perpendicular to the handle surface are measured.

The first step of the algorithm is to solve the equation for the static equilibrium condition represented in Figure 3a in order to find the reaction force $\vec{R}$ (Equation (1)), which is equal to the push force in the opposite direction (${\vec{F}}_{p}=-\vec{R}$).

(1) $$\sum_{i=1}^{n}{\vec{F}}_{i}+\vec{R}=0$$

With the reaction force $\vec{R}$ calculated from the measurements, the minimization algorithm is then applied as follows:

(2) $$\underset{w}{\mathrm{Min}}\sum_{i=1}^{n}{\left(w_{i}||{\vec{F}}_{i}||-\frac{1}{n}\sum_{i=1}^{n}\left(w_{i}||{\vec{F}}_{i}||\right)\right)}^{2}$$

Subject to:

(3) $$\sum_{i=1}^{n}\left(w_{i}{\vec{F}}_{i}\right)=-\vec{R}$$

(4) $$w_{i}\geq 0$$

(5) $$w_{i}\leq 1$$

(6) $$w_{i}{\vec{F}}_{i}\cdot \vec{R}\leq 0$$

The minimization algorithm maximizes the spread of the force (minimizing the variance) contributing to the pushing of the tool ($w_{i}||{\vec{F}}_{i}||$), subject to the static equilibrium condition (Equation (3)) and ensures that only push forces can be applied in the direction normal to the handle’s surface (Equations (4)–(6)).

From the proportion $w_{i}$, the push and the grip force distributions can be computed. From the two distributions, the resulting push force ${\vec{F}}_{p}$, the grip force in the X direction $F_{g-X}$, and the grip force in the Y direction $F_{g-Y}$, can be computed as shown in Figure 3 (see Appendix A for details). The push force in both directions, $F_{p-X}$ and $F_{p-Y}$, were defined as the component of ${\vec{F}}_{p}$ in X and Y, respectively.

### 2.4. Algorithm Validation Method

**Figure 4:**
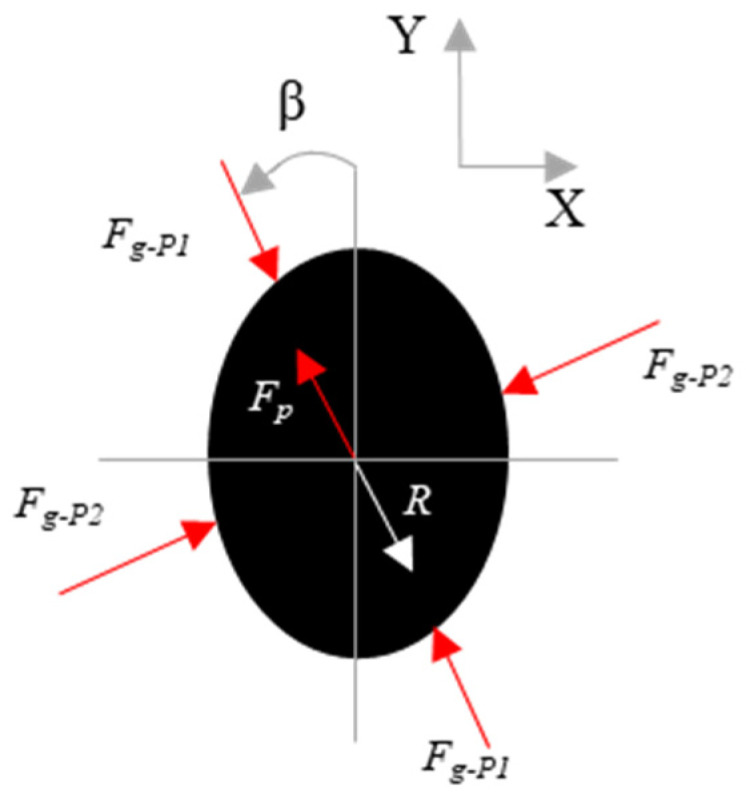
First principal grip force and the rotation of the reference coordinate system.

To validate the algorithm, the error of the reconstructed reaction forces ($\left||\sum_{i=1}^{n}\left(w_{i}{\vec{F}}_{i}\right)+\vec{R}|\right|$) was first computed for every bolt. Second, in order to understand the accuracy of the grip force estimates, the principal grip force was computed, which was defined as the rotation of the reference coordinate system by β, such that $F_{g-P1}$ is maximal [13] (see Figure 4). The β value, referred as the first principal angle, was then compared with other works where only grip forces were applied.

The resultant hand contact force $F_{c}$, defined as the sum of the norm of the distributed normal force at the hand-handle interface surrounding the handle ($\sum_{i=1}^{n}||\vec{F_{i}}||$) was then computed. This contact force has been shown to be linear in $F_{p-Y}$, and $F_{g-Y}$ for circular handles [10,15]. A least square estimation was applied in order to find the linear coefficients that best estimated the contact force (Equation (7)).

(7) $$\hat{F_{c}}=aF_{g-Y}+bF_{p-Y}$$

The minimization problem (Equation (2)) was then recomputed to maximize the variance (instead of minimizing it) in order to observe the effect of the cost function on the grip force distribution. The gripping contact force, defined as the sum of the norm of the grip force distribution at the hand-handle interface, was then computed to observe any difference across the participants as follows:

(8) $$F_{c-g}=\sum_{i=1}^{n}\left(||{\vec{F}}_{i-g}||\right)$$

### 2.5. Data Analysis

The mean force data while the trigger was engaged were analyzed in this paper. The means of six trials for every task for each participant were computed to visualize the effect of the task on the grip and push forces. A subject-specific Pearson correlation coefficient was computed between the contact grip force and the contact push force in order to understand the coupling between the two forces. The contribution of the grip force to the total force ($F_{c-g}/F_{c}$) was computed and compared with the computed value from the coupling force definition ($F_{grip-Y}/F_{coupling}$) [23,24], where:

(9) $$F_{coupling}=F_{g-Y}+F_{p-Y}$$

Friedman tests (α = 0.05) were used to identify any statistical difference at the group level (comparing more than two distributions). If a difference was observed at the group level, the Friedman test was followed by Wilcoxon signed rank tests (*p* = 0.05) with Bonferroni corrections to identify any statistical differences between the tasks/participants. Those statistical tests are non-parametric, and there was no underlying assumption regarding the distributions of the tested samples.

## 3. Results

**Figure 5:**
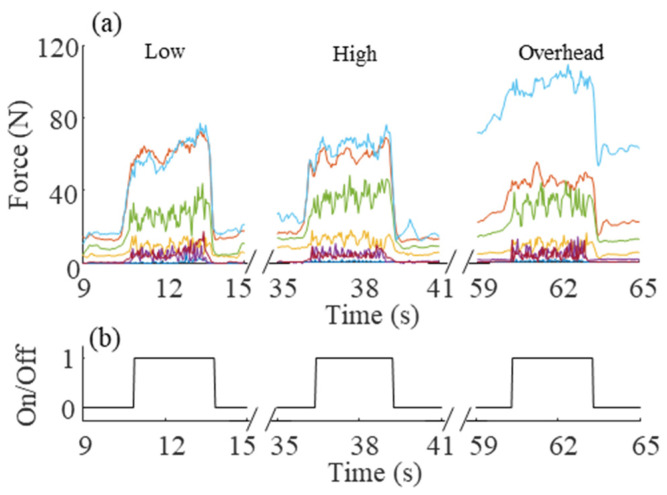
(**a**) Force measurements in the XY plane, and (**b**) on/off signal of the tool trigger for a low, high, and overhead task. Force measurements are color-coded according to Figure 3.

A representative participant’s measured forces are shown in Figure 5 along with the tool trigger engagement data for a low, high, and overhead task.

### 3.1. Algorithm Validation Results

The error of the reconstructed reaction force ($\left||\sum_{i=1}^{n}\left(w_{i}{\vec{F}}_{i}\right)+\vec{R}|\right|$) was computed for every bolt fastening. The data from two participants were removed in the overhead task (12 fastening total) due to a large reconstruction error of −24.9 ± 7.4 N (mean ± SD). After the removal of those outliers, the error of the reconstructed reaction force was negligible with a mean ± SD of −0.3 ± 0.9 N, −0.4 ± 0.9 N, and −1.5 ± 2.5 N for the low, high, and overhead tasks, respectively.

**Figure 6:**
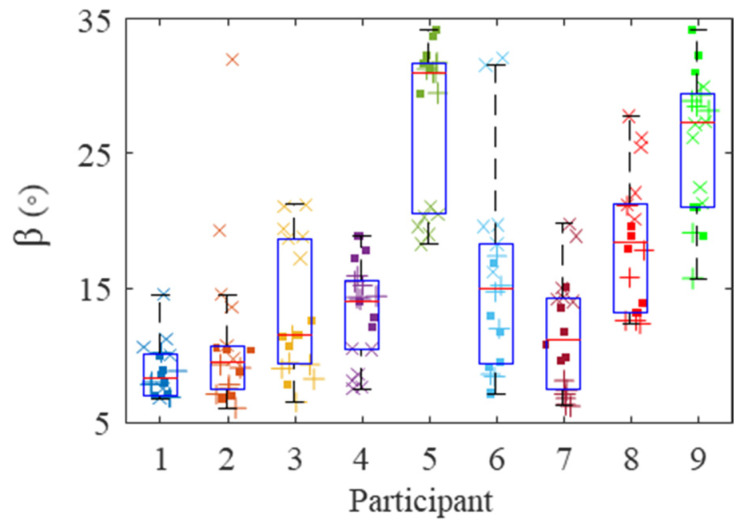
First principal angle for every participant. Each marker represents the mean angle during fastening, where the low, high, and overhead tasks are represented by squares, plus signs, and crosses, respectively.

The first principal angles (β) are shown in Figure 6 for all participants. The values ranged between 6.1° and 34.2°, which agrees well with the literature [13].

**Figure 7:**
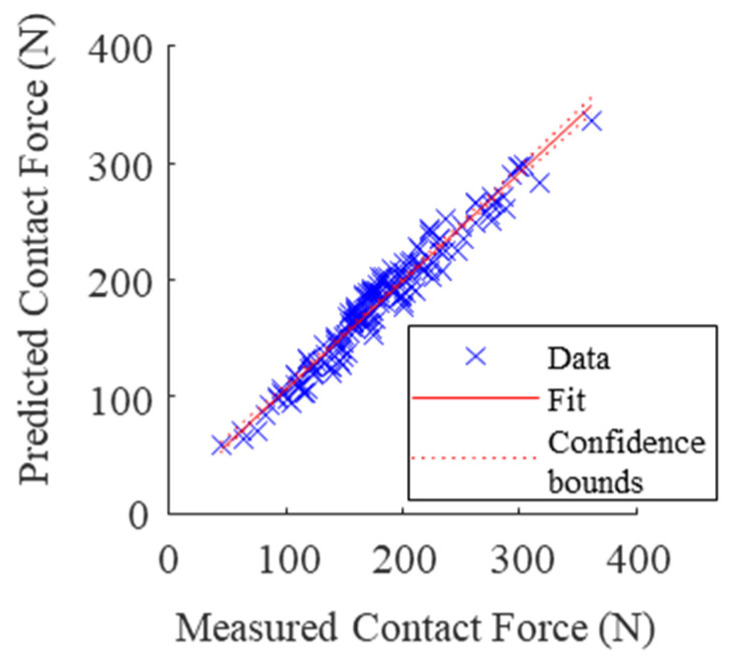
Estimation of contact force using $\hat{F_{c}}$ = 2.51$F_{g-Y}$ + 0.95$F_{p-Y}$, R^2^ = 0.95.

The estimated contact forces from the extracted $F_{p-Y}$ and $F_{g-Y}$ using Equation (7) are shown in Figure 7. The linear coefficients computed by least square were a = 2.51 and b = 0.95. The R^2^ of 0.95 illustrates strong estimation capabilities.

**Figure 8:**
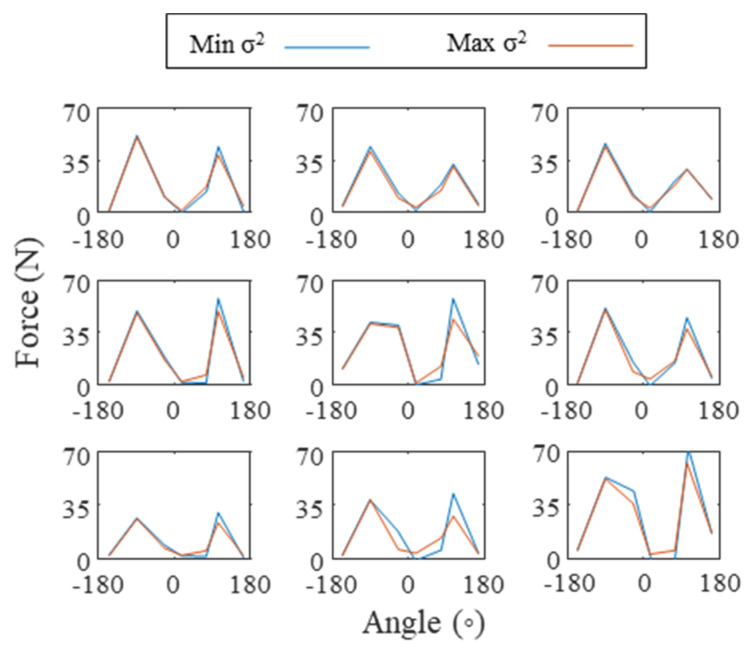
Mean grip force distributions on the tool handle during the low task computed with two different cost functions (minimizing or maximizing the variance of the push force distribution) for the nine participants with order left to right, and top to bottom.

Each participant mean force $||{\vec{F}}_{i-g}||$ as a function of the measurement angle $\theta_{F_{i}}$ for the low task is shown in Figure 8 for the two different cost functions (minimizing or maximizing the variance of the push force distribution). The cost function had a small effect on the actual grip force distribution, which, in turn, had a small effect on the gripping contact force, $F_{c-g}$, of 2.7 ± 4.5% (mean ± SD).

### 3.2. Participants Comparison

**Figure 9:**
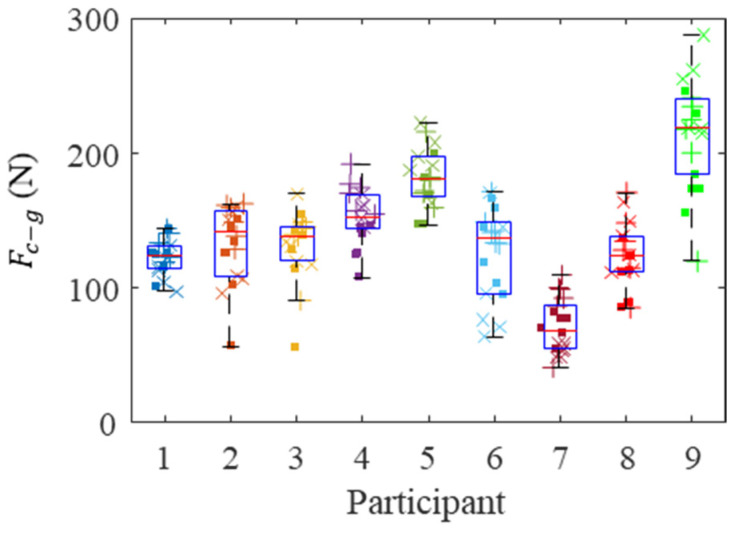
Contact grip forces for every participant. Each marker represents the mean grip force during bolt fastening, where the low, high, and overhead locations are represented by squares, plus signs, and crosses, respectively.

**Figure 10:**
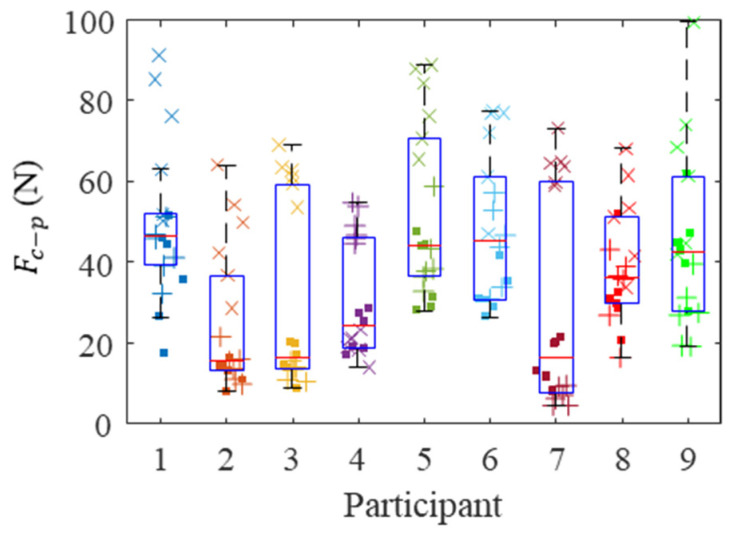
Contact push forces for every participant. Each marker represents the mean push force during bolt fastening, where the low, high, and overhead locations are represented by squares, plus signs, and crosses, respectively.

The gripping contact forces, $F_{c-g}$, and pushing contact forces, $F_{c-p}$, for the nine participants are shown in Figure 9 and Figure 10, respectively. Each marker represents a different bolt, and bolt locations are shown with different marker styles. There was a group-level statistical difference (Friedman test with six repetitions *p* < 0.05) showing a significant difference in both gripping contact forces and pushing contact forces between participants.

The subject-specific Pearson correlation coefficient between the $F_{c-g}$ and $F_{c-p}$ was 0.15 ± 0.48 (mean ± SD) and was 0.06 when using the aggregated data, showing that they were uncorrelated in nature.

### 3.3. Tasks Comparison

**Figure 11:**
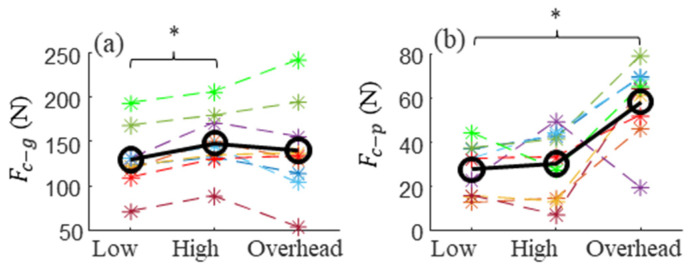
Comparison of the participants’ mean force components: (**a**) the gripping contact force $F_{c-g},$ and (**b**) the pushing contact force $F_{c-p}$ for the different tasks. Each participant is color-coded with respect to Figure 6. Legend: ∗ *p* < 0.05/3.

The participants’ mean contact gripping and pushing forces for each task are shown in Figure 11. The $F_{c-g}$ was significantly higher in the high bolt location in comparison with the low location (Wilcoxon = 0.004). It was observed that every participant increased their $F_{c-g}$ with an increase of 16 ± 9% (mean ± SD). The $F_{c-p}$ was significantly higher in the overhead location in comparison with the low location (Wilcoxon = 0.008). Moreover, if participant number four is considered an outlier, then the $F_{c-p}$ was significantly higher in the overhead location in comparison with the high location (Wilcoxon = 0.008).

**Figure 12:**
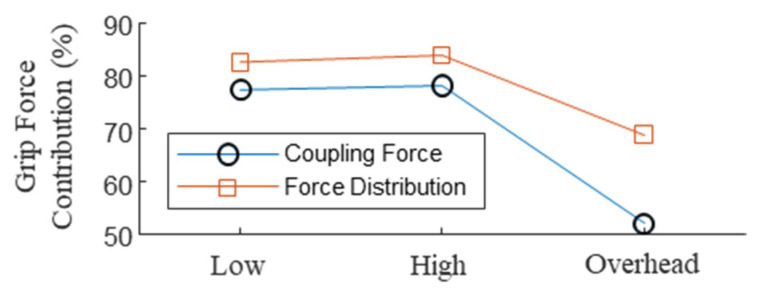
Comparison of the average grip force contribution computed from the force distribution and the coupling force.

The comparison of the grip force contribution to the total hand force when using the coupling force definition ($F_{g-Y}/F_{coupling}$) and our definition using the distributions ($F_{c-g}/F_{c}$) is shown in Figure 12. It shows that the coupling force definition underestimates the grip contribution.

## 4. Discussion

The algorithm presented in this paper enables the quantification of grip and push force distributions on a pistol-grip hand tool from pressure map measurements. To the best of our knowledge, this is a novel demonstration. The isolation of both force distributions on the tool, which is our main contribution, can help to extract the contribution of the grip force (and push force) to the accomplishment of a given task, which was underestimated by previous methods. This can, in turn, be used by ergonomists to better design workstations in assembly lines but could also be used to identify workers who are at a higher risk of injury.

The algorithm was able to reconstruct the push force distribution with low error ($\left||\sum_{i=1}^{n}\left(w_{i}{\vec{F}}_{i}\right)+\vec{R}|\right|$) in 93% of the cases. In the cases where large errors were observed (two participants’ overhead tasks), the push force was largely underestimated due to the constraints of the algorithm. The force measurements that opposed the reaction force were not large enough to cancel it out. This indicates that some forces applied on the tool were not measured. Two hypotheses are to be validated in future studies: (1) This extra force was applied locally between sensors and (2) the friction force cannot be neglected for specific hand grip position. The ability to flag erroneous force reconstruction is a benefit of this algorithm as it is related to measurement issues. This information is not available from the raw data nor from other grip and push force-extraction algorithms [21,22], and therefore, our algorithm can make sure that conclusions are not based on erroneous pressure map measurements. 

Previous work has shown that the first principal angle when gripping a cylindrical tool is 78.2 ± 11.5° (mean ± SD) [13] when referenced about the z_h_-axis of the biodynamic hand coordinate system that is defined in ISO 8727 [12]. The z_h_-axis, though not measured for every participant, is approximately −45° from the Y-axis. Herein, the observed values of the first principal angle (β) ranged between 6.1° and 34.2° about the Y-axis (Figure 6). Therefore, the β are similar to previous works when considering that the handle had an ellipsoidal cross-section.

It was shown that the proposed linear relationship between grip force, push force, and the contact force is still applicable with an ellipsoidal cross-section handle [10] (see Figure 7). The linear coefficients, a and b, in [10] were 3.40 and 0.97, whereas in this work they were 2.51 and 0.95, respectively. The difference in a can be explained by both the difference in handle cross-section (circular vs ellipsoidal) and the orientation of the grip force (Y-axis vs z_h_-axis).

Other than assuming all of the forces applied by the user on the pistol-grip tool are measured, the main hypothesis behind the algorithm is the cost function used. We hypothesized that when a user pushed on the tool handle, the distribution of the pressure on their hand is maximized due to the compliance of the hand. It has to be emphasized that minimizing the variance of the pushing force components, subject to the constraints herein, could only spread the force on half of the handle since half of it was constrained to be 0 N. In the case of seven discrete circumferential measurements, it was shown that the cost function has little impact on the final grip force distribution (Figure 8). Other than visually observing similar results from both cost functions, it was shown that the difference in the computed gripping contact force was 2.7 ± 4.5% (mean ± SD), which would not change the conclusions if a user applied more grip force than required for a given task. The choice of the cost function, however, will be more important with a better pressure sensing resolution. As a counter example, with only four measurements that are 90° apart, there is only one solution to this problem, which is the static equilibrium point. However, as the number of measurements increases, there are more possible solutions to reconstruct the push force. In the case of many measurements, it is hypothesized that minimizing the variance is more representative of the push force distribution, based on the observation in [20] in which the pressure distribution in the thenar region of the hand was fairly constant for a pure pushing action.

One of the main benefits of using the algorithm presented here is to be able to extract the amount of contact grip and push forces between tasks solely from the pressure measurements, as shown in Figure 11. As expected, the amount of push force required to fasten a bolt in overhead location is significantly larger than when fastening in a plane perpendicular to gravity (low and high locations). This statement does not hold for grip force as gravity did not affect the amount of grip force used to accomplish the task. However, fastening at a higher level significantly increased the amount of grip force used compared to the low level, with an increase of 16 ± 9%.

Extracting contact grip and push forces is also helpful when comparing individuals’ performance. As observed in Figure 9 and Figure 10, individuals with large grip forces are not necessarily pushing more. We showed a four-fold difference in median contact gripping force and a significant difference in the contact push force among experienced factory workers. Some amount of counterforce is needed in order to stabilize a hand tool that is applying a given torque. Even if we allow for a small margin of error and personal preference, we hypothesized that assembly line workers would have arrived at this minimum counterforce needed by experience and hence, only a small variation in grip force was anticipated. Our study demonstrated that a significant variation in grip force exists even among experienced automobile assembly line workers. A similar study reported a large variation in palmar grip forces [25]. We hypothesized that as a worker became more experienced using a power tool, they would relax their grip to a comfortable, minimum level. The results of our study showed that this hypothesis was incorrect. Workers with even significant power tool experience applied more grip force than was required for a task. Therefore, by using the algorithm, the appropriate corrective measure can be tailored to the individual when excessive force is applied.

It was shown that for the fastening tasks tested herein, the grip force had a larger contribution to the overall hand force than the pushing action (Figure 12). This was shown in both [15,21], where cumbersome equipment was needed to arrive at the same conclusion. Moreover, the model that was used was handle- and subject-specific. With our algorithm, the extraction of both grip and push force contributions to the hand contact force can be done solely with a pressure mapping sensor. The coupling force definition approach [23,24] can be used without the algorithm; however, it largely underestimates the contribution of the grip to the total hand force, since it does not consider the distribution of both forces.

The main limitation of this study is the lack of validation of the computed push and grip forces using an instrumented handle and/or a force plate. However, based on [13,21], a small but acceptable error of the computed push and grip forces are expected when derived from pressure mapping sensors. Future steps should include a force plate to validate/calibrate the push force when performing real work-like tasks. Future steps should also include more participants in order to study sex differences, different handle sizes in order to adapt to the participants’ hand span, and different fasten heights in order to study the impact of tool angles on grip and push forces. Moreover, sensory tests should be performed in order to include sensory deficit as a factor impacting grip and push forces.

It is well known that forceful exertion is directly related to UECTD; therefore, it is important to reduce the unnecessary gripping force that is applied during the hand tool operation by monitoring hand-handle contact forces. New assembly line workers can experience high levels of muscle fatigue by applying grip force far beyond what is required for the task, while experienced workers can be set in their way of over-gripping, further increasing their risk of injury as they age. This is especially relevant when considering non-occupational UECTD risk factors given North America’s aging and overweight workforce. Werner et al. found that automotive and industrial workers over 40 years old have a 76% increased risk of upper extremity tendonitis, while those classified as obese (BMI > 30) are 93% more likely to be diagnosed with CTS [26]. With an increasing average age and obese percentage of the work force, it is important to devise measures to educate the workforce, implement effective training programs, and monitor those at a higher risk of injury. Practitioners can improve the health and well-being of workers while reducing the costs associated with lost work hours by minimizing the risk of UECTDs that are caused by over-gripping.

## 5. Conclusions

In this paper, an algorithm is proposed to isolate the measured force distribution on a handle into distinct grip and push force distributions, in contrast to other available methods, which decouples grip and push into single force vectors. The contributions of this paper are two-fold. First, we demonstrated the feasibility of separating the grip and push force distributions from the measured hand-handle force distribution, which can provide salient information on the task being performed. Second, the collected data on low, high, and overhead fastening tasks with a pistol-grip electric nutrunner showed a large variability in grip and push forces across tasks, as well as a large variability between individuals. Moreover, the uncorrelated nature of the grip and push forces illustrated the need to decouple the force measurements in order to accordingly minimize the risk of injuries.

## Figures and Tables

**Table 1:** Participant information summary.

	Female		Male	
	Mean	±SD	Mean	±SD
**Height, cm (in)**	160.3 (63.1)	3.4 (1.3)	178.3 (70.2)	10.9 (4.3)
**Hand Span, cm (in)**	18.5 (7.3)	0.8 (0.3)	20.7 (8.1)	1.0 (0.4)
**Age, years**	48.5	2.3	40.0	10.1
**Power Tool Experience, years**	10.6	6.9	17.4	7.1
**Employed at Manufacturer, years**	23.5	4.5	14.0	8.5

## Data Availability

The data may be requested from N.C. (nchandra@uwaterloo.ca).

## Appendix A

From the proportion $w_{i}$, the push and the grip force distributions are computed, respectively, as follows:

(A1) $${\vec{F}}_{i-p}=-w_{i}{\vec{F}}_{i}$$

(A2) $${\vec{F}}_{i-g}={\vec{F}}_{i}-w_{i}{\vec{F}}_{i}$$

The grip force in both directions, $F_{g-X}$ and $F_{g-Y}$, are computed as follow (see Figure 3a):

(A3) $$F_{g-X}=\frac{1}{2}\sum_{i=1}^{n}\left({\vec{F}}_{i-g}\cos\theta_{i}\right)$$

(A4) $$F_{g-Y}=\frac{1}{2}\sum_{i=1}^{n}\left({\vec{F}}_{i-g}\sin\theta_{i}\right)$$
